# Fuc-S—A New Ultrasonic Degraded Sulfated α-l-Fucooligosaccharide—Alleviates DSS-Inflicted Colitis through Reshaping Gut Microbiota and Modulating Host–Microbe Tryptophan Metabolism

**DOI:** 10.3390/md21010016

**Published:** 2022-12-25

**Authors:** Haitao Xiao, Jinxiu Feng, Jiao Peng, Peigen Wu, Yaoyao Chang, Xianqian Li, Jinhui Wu, Haifeng Huang, Huan Deng, Miao Qiu, Yuedong Yang, Bin Du

**Affiliations:** 1 School of Pharmaceutical Sciences, Health Science Center, Shenzhen University, Shenzhen 518060, China; 2 Hebei Key Laboratory of Natural Products Activity Components and Function, Hebei Normal University of Science and Technology, Qinhuangdao 066004, China; 3 Department of Pharmacy, Peking University Shenzhen Hospital, Shenzhen 518036, China; 4 School of Pharmaceutical Sciences, Guizhou Medical University, Guiyang 550031, China

**Keywords:** fucoidan, ultrasonic degradation, colonic inflammation, gut microbiota, tryptophan metabolism

## Abstract

Scope: The dysbiosis of intestinal microecology plays an important pathogenic role in the development of inflammatory bowel disease. Methods and Results: A polysaccharide named Fuc-S, with a molecular weight of 156 kDa, was prepared by the ultrasonic degradation of fucoidan. Monosaccharide composition, FTIR, methylation, and NMR spectral analysis indicated that Fuc-S may have a backbone consisting of →3)-α-L-Fucp-(1→, →4)-α-L-Fucp-(1→ and →3, 4)-α-D-Glcp-(1→. Moreover, male C57BL/6 mice were fed three cycles of 1.8% dextran sulfate sodium (DSS) for 5 days and then water for 7 days to induce colitis. The longitudinal microbiome alterations were evaluated using 16S amplicon sequencing. In vivo assays showed that Fuc-S significantly improved clinical manifestations, colon shortening, colon injury, and colonic inflammatory cell infiltration associated with DSS-induced chronic colitis in mice. Further studies revealed that these beneficial effects were associated with the inhibition of Akt, p-38, ERK, and JNK phosphorylation in the colon tissues, regulating the structure and abundance of the gut microbiota, and modulating the host–microbe tryptophan metabolism of the mice with chronic colitis. Conclusion: Our data confirmed the presence of glucose in the backbone of fucoidan and provided useful information that Fuc-S can be applied as an effective functional food and pharmaceutical candidate for IBD treatment.

## 1. Introduction

Inflammatory bowel disease (IBD) is a kind of chronic inflammatory condition of the intestinal mucosa with two subtypes—ulcerative colitis (UC) and Crohn’s disease (CD)—characterized by dysregulation of the tightly regulated intestinal immune system [1,2]. Traditionally, IBD is mostly found in North America and Europe [3]. Currently, IBD represents a global health problem with increasing incidence in industrialized countries, which not only seriously affects the quality of life of patients but also poses a substantial burden to healthcare systems [2]. So far, the exact pathogenesis of IBD has not been well clarified, triggered by the interplay of genetic susceptibility, immune responses, intestinal microflora, and environmental factors; in consequence, there is no curative treatment [4]. Thus, significant research efforts to improve the treatment are urgent to address this unmet need.

In clinical practice, current strategies for the treatment of IBD largely focus on inducing remission and preventing relapse [5], including corticosteroids, aminosalicylates, biologic agents, and immunosuppressants. These traditional therapeutic drugs cannot achieve satisfactory results because of their side effects with long-term use [6]. Recently, a lot of studies have investigated foods sourced from natural products that exhibit the potential to help induce remission of IBD, increasingly attracting the attention of researchers to develop nutraceuticals as new adjuvant therapies for IBD [7,8,9,10,11,12]. Fucoidan—a sulfated polysaccharide extracted from brown seaweed and other marine lifeforms—is an ideal selected target, as it is non-toxic and its beneficial effects on inflammatory diseases, neurodegenerative diseases, oxidative stress, and tumors have been well demonstrated by many different reports, both in vivo and in vitro [13,14,15]. This evidence suggests the fucoidan could be a therapy option for IBD. However, commercial fucoidans usually contain multiple peaks of molecular weight (Mw), causing their activities to vary greatly. The homogeneous bioactive fucoidans were prepared and purified, with important application value for health promotion. Fucoidans are complex polysaccharides derived from brown seaweeds that consist of considerable proportions of L-fucose and other monosaccharides, along with sulfated ester residues [16]. Usoltseva et al. [17] established the fine structure of fucoidan from *Sargassum oligocystum*. The fucoidan from *S. oligocystum* was sulfated (32%) galactofucan (Fuc:Gal = 2:1) with an Mw of 183 kDa (Mw/Mn = 2.0). Its supposed structure was found to be predominantly 1,3-linked fucose as the main chain, with branching points at C2 and C4. In another study, a fucoidan with a novel structure was extracted from *Sargassum fusiforme*. It had a molecular weight of 703 kDa and was composed of fucose and galactose at a ratio of 73.16:26.84 (mol%). Structural analyses showed that it mainly consisted of 1,3-, 1,4-, and 1,3,4-linked-α-l-Fucp and 1,3-, 1,6-linked-β-d-Galp, with partial sulfation of fucose units at C-4 and C-3, and of galactose units at C-6 and C-3 [18].

As we know, partial depolymerization of high-molecular-weight polysaccharides into low-molecular-weight or medium-molecular-weight polysaccharides with high-intensity ultrasound is a strategy to improve their solubility, homogeneity, and biological activities [13,19,20]. Ultrasonic treatment is considered to be one of the most promising, effective, and environmentally friendly physical treatments for preparing fractions with different molecular weights, as it allows the degraded products to be more easily recovered and purified with no change in structure. Compared with acid hydrolysis, alkaline hydrolysis, γ-irradiation, or enzyme hydrolysis, ultrasonication technology is a good physical method for producing homologous series with lower molecular weights. Recently, ultrasonication has been widely applied for the controllable degradation of natural polysaccharides such as *Cordyceps* exopolysaccharide [21], konjac glucomannan [22,23,24], fucoidan and *Ganoderma lucidum* beta-glucan [25], resulting in changes in solution properties and biological properties.

The human gut harbors a complex and abundant aggregation of microbes, collectively referred to as the gut microbiota. The gut microbiota has physiological functions associated with nutrition, the immune system, and defense of the host (Nishida et al., 2018). Moreover, microbial imbalance (also called dysbiosis) is associated with IBD.

Herein, we developed an ultrasonication method to prepare homogeneous polysaccharides from heterogeneous fucoidans and reported that a new homogeneous polysaccharide named Fuc-S, with high Mw, was degraded from the heterogeneous fucoidan produced from *Fucus vesiculosus*. Monosaccharide composition, molecular weight analysis, Fourier-transform infrared (FTIR) spectroscopy, methylation, and nuclear magnetic resonance (NMR) spectral analysis were also carried out, and its anti-colitis activity was evaluated in DSS-challenged chronic colitis. The favorable therapeutic effects of Fuc-S on colonic inflammation indicate that it may be a potential nutraceutical candidate for the treatment of IBD.

## 2. Results

### 2.1. Structural Characterization of Fuc-S

#### 2.1.1. Mw and Chemical Composition Analysis

**Figure 1:**
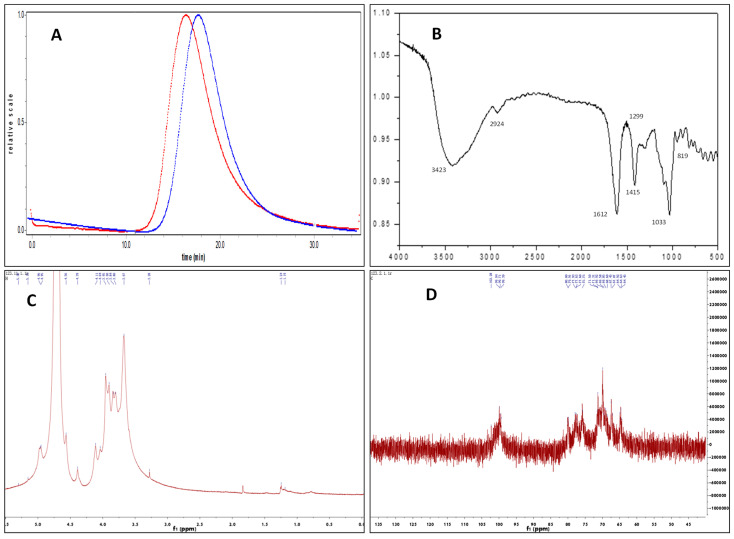
Characterization of Fuc-S by homogeneity, FTIR, and 1D-NMR spectral analysis: (**A**) HPSEC elution profile of Fuc-S, detected with a multi-angle laser light scattering detector (blue) and refractive index detector (red). (**B**) FTIR spectrum of Fuc-S. (**C**) ^1^H-NMR spectrum of Fuc-S. (**D**) ^13^C-NMR spectrum of Fuc-S.

As shown in Figure 1A, a single symmetrical peak in the chromatograph of HPSEC suggested that Fuc-S is a homogeneous polysaccharide. The Mw of Fuc-S was determined to be 156 kDa by HPSEC-MALLS analysis, and its polydispersity was calculated to be 1.42, suggesting that Fuc-S has a broad molecular weight distribution. Additionally, the slope between the radius of gyration (Rg) and the Mw of Fuc-S was calculated to be 0.49, indicating that Fuc-S existed in aqueous solution as a random coil (Please check Supplementary Material, Figure S1). Monosaccharide analysis revealed that Fuc-S consisted of fucose, glucose, galactose, xylose, rhamnose, mannose, galacturonic acid, and glucuronic acid, at a molar ratio of 0.49: 0.28: 0.12: 0.01: 0.02: 0.04: 0.01:0.02 (Figure S2). 

#### 2.1.2. FTIR Spectral Analysis

In the FTIR spectra of Fuc-S (as shown in Figure 1B), the peak at 3423 cm^−1^ was the stretching vibration of –OH [26,27]. The signal at around 1612 cm^−1^ was produced by the bending vibrations of H-OH [26,27,28,29]. The absorption peaks at approximately 2924 and 1415 cm^−1^ were the C-H stretching vibration and bending vibration of free sugars, respectively [30,31]. In particular, the band at 1299 cm^−1^, corresponding to the asymmetric O=S=O stretching vibration, indicated the presence of sulfate [29,32]. The band at around 1033 cm^−1^ showed the stretching vibration of C-O-C glycosidic bonds [30,31,32,33], and the peaks at around 819 cm^−1^ were assigned to the mannose residues coinciding with the published composition analysis [34,35].

#### 2.1.3. Methylation Analysis

On the basis of standard data and retention time in the Complex Carbohydrate Research Center (CCRC) spectral database, the ratios of the methylated alditol acetates of Fuc-S were identified, as listed in Table 1. The fucose-based sugar residues (1,3-linked Fucp and 1,4-linked Fucp, with proportions of 0.43 and 0.15, respectively), glucose-based sugar residues (1,3,4-linked Glcp and 1,2,4,6-linked Glcp, with proportions of 0.11 and 0.10, respectively), and galactose sugar residues (the proportions of 1,6-linked Galp and 1,3,4,6-linked Galp were 0.06 and 0.07, respectively) were highly enriched in Fuc-S, suggesting that 1,3-linked Fucp and 1,4-linked Fucp were the main components of the backbone of Fuc-S. Furthermore, only 1,3-linked Fucp and 1,4-linked Fucp were detected, suggesting that polysubstituted Glcp and/or Galp was also the main component(s) of the backbone of Fuc-S to build the branch chain(s).

#### 2.1.4. NMR Analysis

**Figure 2:**
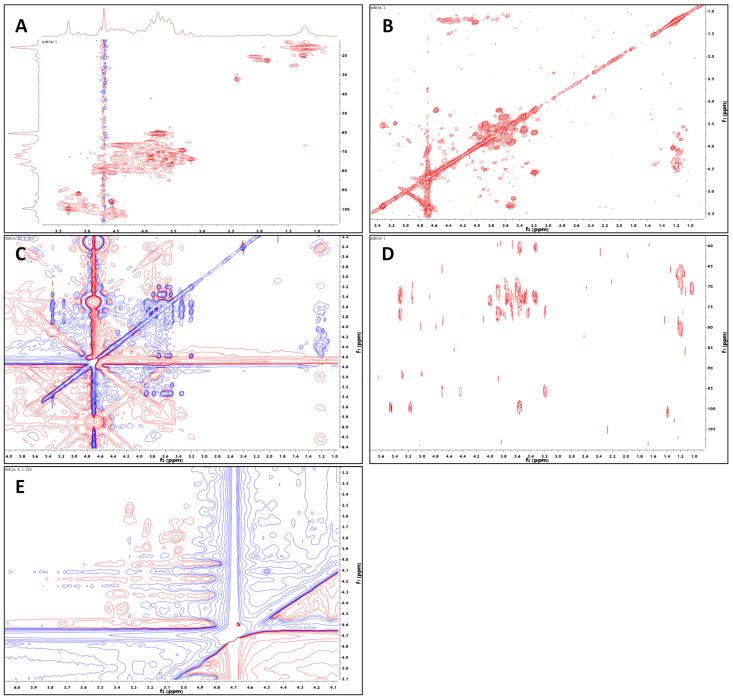
2D-NMR spectra of Fuc-S: (**A**) HSQC, (**B**) ^1^H-^1^H COSY, (**C**) ^1^H-^1^H TOCSY, (**D**) HMBC, (**E**) ^1^H-^1^H ROESY.

Deep specific structural characteristics of Fuc-S were identified by ^1^H, ^13^C NMR, and 2D-NMR (COSY, TOCSY, HSQC, HMBC, and ROESY) spectra (Figures S3–S6). As shown in Table 2, Figure 1, and Figure 2, the ^1^H, ^13^C, and HSQC spectra indicated that Fuc-S was mainly composed of six kinds of residues (denoted as residues A–F), with their anomeric proton signals at δ 5.03 (98.27), 5.22 (99.79), 5.32 (99.62), 4.96 (100.32), 5.15 (91.89), and 4.57 (95.88), respectively. Non-anomeric sugar protons were obtained in the 1.10–4.52 ppm region. The two peaks at 1.19 and 1.24 ppm were typical proton signals of methyl protons of fucopyranose residues, and one-proton signals at 4.36 ppm in the low-field region indicated that one-sulfate sulfatases were present in the sugar residues, while another proton signal at 4.52 ppm in the low-field region indicated that another sulfate sulfatase was present in the sugar residues. In addition, the signals at about δH 1.88 and δC 22.57 along with δC 179.02, and at δH 2.42 and δC 32.41 along with δC 180.10, indicated two O-acetyl groups linked to the sugar residues.

For residues A and B, the chemical shifts of protons and carbon could be assigned with the aid of HSQC, COSY, TOCSY, and HMBC (as shown in Table 2). For residue A, the TOCSY peaks at δ 5.03/3.78, 5.03/3.94, 1.18/3.94, 1.18/4.36, and 1.18/4.08 ppm, COSY peaks at δ 5.03/3.78, 3.94/3.78, 3.94/4.36, 4.08/4.36, and 4.08/1.18 ppm, and HMBC peaks at 3.94/68.94, 3.94/79.66, 3.78/76.06, 4.08/79.66, and 1.18/66.68 ppm identified residue A as →3)-α-L-Fucp(4SO_4_^2−^)-(1→, according to the results of the methylation analysis and other literature [36,37]. For residue B, the cross-peaks at δH 5.22/4.02, 5.22/3.89, 5.22/3.72, 4.02/3.89, and 4.02/3.79 ppm in the TOCSY spectrum, along with the cross-peaks at δH 5.22/3.72, 3.72/3.89, 3.89/3.79, 4.02/3.79, and 4.02/1.24 ppm in the COSY spectrum, indicated H1/C1-H6/C6 signals of residue B. Furthermore, these assignments could be observed by the cross-peaks at δ 3.72/73.06, 3.89/68.39, 3.89/77.75, 3.79/68.22, 4.02/77.75, and 1.24/80.02 ppm in the HMBC spectrum. According to the methylation analysis and previous reports [29,38], residue B was elucidated as →4)-α-L-Fucp-(1→.

Similar to residues A and B, the TOCSY peaks at δH 5.15/4.11, 5.15/3.76, 5.15/3.89, 5.15/3.58, and 5.15/3.50 ppm, COSY peaks at δH 5.15/3.50, 3.89/3.50, 3.89/4.11, 3.58/4.11, and 3.58/3.76 ppm, and HMBC peaks at δH 3.50/76.29, 3.89/71.45, 3.89/77.35, 4.11/76.29, 4.11/70.06, 3.76/70.06, and 3.58/60.31 indicated residue C as →3, 4)-α-D-Glcp-(1→, according to the methylation results and previous reports. In parallel, the TOCSY correlations of δH 5.32/3.91, 5.32/3.77, 5.32/3.59, 5.32/3.54, and 5.32/3.30 ppm, COSY correlations of δH 5.32/3.54, 3.91/3.54, 3.91/3.77, 3.59/3.77, and 3.59/3.30, and HMBC correlations of δ 3.30/72.62, 3.59/69.24, 3.59/71.26, 3.77/72.62, 3.91/71.26, 3.91/71.46, and 3.54/72.62 ppm also indicated residue D as →6)-α-D-Galp-(1→.

Similarly, combined with the data of methylation and NMR signals, residue E was identified as →2, 4, 6)-α-d-Glcp(3SO_4_^2−^)-(1→, based on the TOCSY correlations of δH 4.96/3.37, 4.96/3.61, 4.96/3.89, and 4.96/3.76 ppm, the COSY correlations of δH 4.96/3.88, 4.50/3.88, 3.88/3.58, 3.75/3.58, and 3.75/3.32 ppm, and the HMBC correlations of δ 3.32/71.21, 3.58/71.21, 3.75/76.75, 3.75/69.24, 3.58/80.70, and 4.52/77.34 ppm. In parallel, residue F was identified as →3, 4, 6)-β-d-Galp-(1→, with the TOCSY correlations of δH 3.20/4.57, 3.53/4.57, 3.58/4.57, 3.69/4.57, and 3.20/3.80 ppm, the COSY correlations of δH 4.57/3.20, 3.69/3.20, 3.69/3.80, 3.80/3.58, and 3.58/3.53 ppm, and the HMBC correlations of δ 3.20/76.13, 3.20/95.88, 3.69/76.82, 3.80/76.13, 3.53/72.74, and 3.58/71.50 ppm.

The glycosidic linkage of Fuc-S was identified with the aid of ROESY and HMBC. In the ROESY spectrum, the correlations of H1 (δH 5.03) of residue A to H4 (δH 3.80) of residue B, and of H1 (δH 5.22) of residue B to H3 (δH 3.89) of residue C, indicated the existence of →3)-A-(1→4)-B-(1→3)-C-(1→ linkage. In parallel, the ROESY correlation of H1 (δH 5.03) of residue A to H3 (δH 3.94) of residue A, along with the HMBC correlations of H1 (δH 5.03) to C3 (δC 76.06) of residue A, indicated the existence of repeated →3) -A-(1→3)-A-(1→ linkages. Moreover, the HMBC correlations of H3 (δH 3.94) of residue A to C1 (δC 91.89) of residue C, and of H1 (δH 5.15) of residue C to C3 (δC 76.06) of residue A, indicated the existence of →3)-C-(1→3)-A-(1→ linkages. Based on the molar ratio of the methylation analysis, the backbone of Fuc-S could be deduced as →3-A-(1→3)-A-(1→3)-A-(1→4)-B-(1→3)-C-(1→3)-A-(1→3)-A-(1→3)-A-(1→4)-B-(1→3)-C-(1→. Clearly, except for the backbone, two branches were built at the C-4 position of residue C. Interestingly, the HMBC correlations of H4 (δH 4.10) of residue C to C1 (δC 100.32) of residue E, and of H1 (δH 4.96) of residue E to C4 (δC 77.35) of residue C, indicated the existence of -C-(4→1)-E- linkages. In addition, the ROESY correlation of H1 (δH 5.32) of residue D to H4 (δH 3.58) of residue E indicated the existence of -D-(1→4)-E- linkages. In parallel, the HMBC correlations of H2 (δH 3.88) of residue E to C1 (δC 95.88) of residue F, and of H1 (δH 4.57) of residue F to C2 (δC 77.34) of residue E, indicated the existence of -F-(1→2)-E- linkages. Finally, two weak HMBC correlations of δH 3.30 with δC 179.02 and of δH 3.51 with δC 180.10 indicated the existence of two O-acetyl groups that were linked to the C-6 positions of residues D and F, respectively. Collectively, the possible main structure of Fuc-S was assumed to be the following repeating unit (Figure S8).

### 2.2. Anti-Colitis Effects of Fuc-S on DSS-Induced Chronic Colitis in Mice

#### 2.2.1. Fuc-S Attenuates DSS-induced Chronic Colitis in Mice

**Figure 3:**
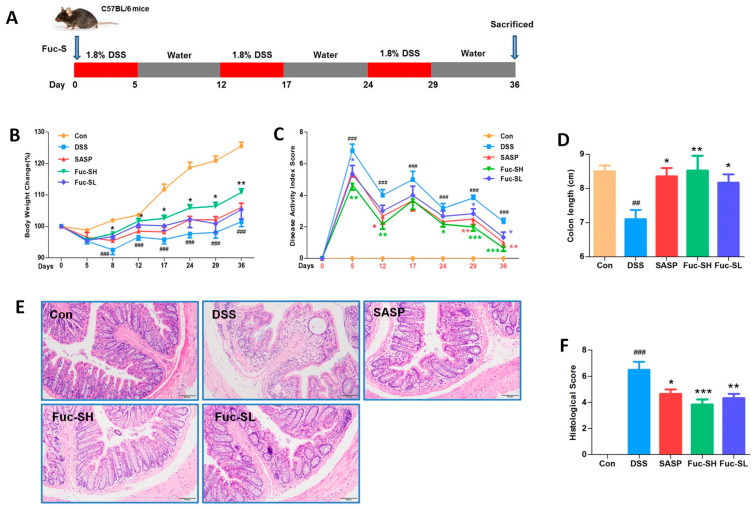
Fuc-S improved clinical manifestations and colon injury of DSS-induced chronic colitis in mice: (**A**) Flowchart of the experiment and treatment. (**B**) Effect of Fuc-S on body weight loss. (**C**) Effect of Fuc-S on disease activity index (DAI). (**D**) Effect of Fuc-S on colon shortening. (**E**,**F**) Effect of Fuc-S on colon damage. Scale in (**E**): 100 μm. Results are expressed as the mean ± SEM (*n* = 6) and were analyzed using ANOVA followed by Duncan’s multiple range test; ## *p* < 0.01, and ### *p* < 0.001, compared to the Con group; * *p* < 0.05, ** *p* < 0.01, and *** *p* < 0.001, compared to the DSS group. Con: control, SASP: salazosulfadimidine, Fuc-SH: Fuc-S with high dosage (200 mg/kg), Fuc-SL: Fuc-S with low dosage (100 mg/kg).

DSS-induced chronic colitis is a technically mature preclinical model with many phenotypic features similar to human IBD [39]. In this model, significant weight loss and colon shortening are two typical clinical symptoms. As shown in Figure 3B, the weight loss and colon shortening induced by DSS were profoundly rescued by Fuc-S (Figure 3D). The DAI score is a responsible marker to assess the severity of colitis, which is calculated by the scores of weight loss change, the incidence of diarrhea, and rectal bleeding, as described in a previous report [6]. As shown in Figure 3C, the DAI score in the DSS group was profoundly elevated, while its elevation was significantly suppressed by the treatment with Fuc-S. In addition, histopathological analysis by hematoxylin and eosin (H&E) staining was carried out to assess the effect of Fuc-S on DSS-induced colon damage. As shown in Figure 3E, the DSS treatment resulted in extensive colonic tissue damage, including inflammatory cell infiltration, lesion formation, and crypt destruction. Mice receiving Fuc-S (200 and 100 mg/kg) treatment had less colon damage with lower scores of histological changes (Figure 3F). In this study, dose selection was based on pre-experiments and references. Interestingly, compared with the SASP-treated group, Fuc-S (200 mg/kg) exerted better protective effects. These results indicate that Fuc-S treatment can rescue the clinical symptomatic features and colon injury of DSS-induced chronic colitic mice.

#### 2.2.2. Fuc-S Attenuates Inflammatory Cell Infiltration and Pro-Inflammatory Cytokine Production in the Colon Tissues of DSS-Induced Chronic Colitic Mice

**Figure 4:**
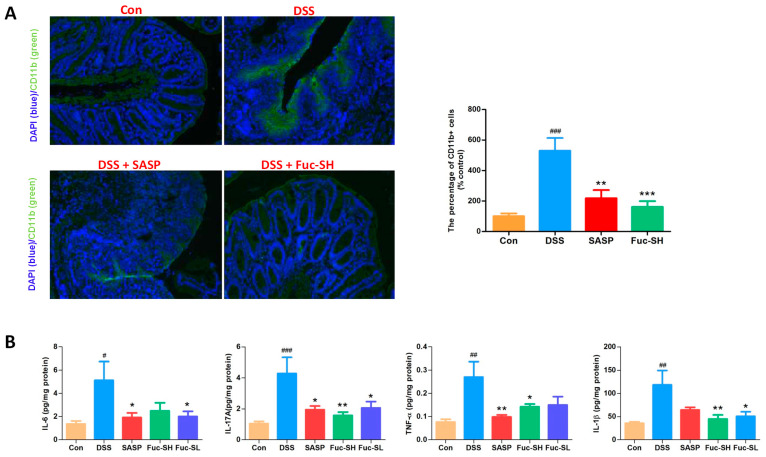
Fuc-S attenuated colonic inflammatory cell infiltration and pro-inflammatory cytokine production of mice with DSS-induced chronic colitis: (**A**) Effect of Fuc-S on CD11b+ inflammatory cell infiltration. (**B**) Effects of Fuc-S on TNF-α, IL-1β, IL-6, and IL-17A production in the colon tissues. Results are expressed as the mean ± SEM (*n* = 6) and were analyzed using ANOVA followed by Duncan’s multiple range test; # *p* < 0.05, ## *p* < 0.01, and ### *p* < 0.001, compared to the Con group; * *p* < 0.05, ** *p* < 0.01, and *** *p* < 0.001, compared to the DSS group. Con: control, SASP: salazosulfadimidine, Fuc-SH: Fuc-S with high dosage (200 mg/kg), Fuc-SL: Fuc-S with low dosage (100 mg/kg).

Infiltration of inflammatory cells to the inflamed colon tissue is an important pathological feature of IBD [40]. CD11b is mainly expressed in monocytes, neutrophils, macrophages, and other leukocytes, and it is usually used as a marker to monitor inflammatory cell infiltration [4]. To further investigate the protective activity of Fuc-S on DSS-induced inflammatory cell trafficking into the colon, immunofluorescence staining was performed with CD11b antibodies. As shown in Figure 4A, a large number of CD11b-positive cells were accumulated in the mucosal lesions of DSS-induced colitic mice, while the number of infiltrating CD11b-positive cells was decreased significantly after Fuc-S treatment. Inflammatory cells accumulated in the inflamed colon tissue were activated, leading to excessive production of pro-inflammatory cytokines to exacerbate colonic inflammation [41]. Consistently, levels of the pro-inflammatory cytokines TNF-α, IL-1β, IL-6, and IL-17A in colonic homogenates of DSS-treated mice were sharply increased in comparison with those of normal control mice, while these increases were also reduced dramatically after Fuc-S treatment (Figure 4B). The present results clearly show that Fuc-S exerts an effective inhibitory effect against inflammatory cell infiltration and pro-inflammatory cytokine production in the colon tissues of DSS-induced chronic colitic mice.

#### 2.2.3. Transcriptomics Analysis Showed That Fuc-S Attenuates Colonic Inflammation by Regulating the PI3K-Akt and MAPK Signaling Pathways

**Figure 5:**
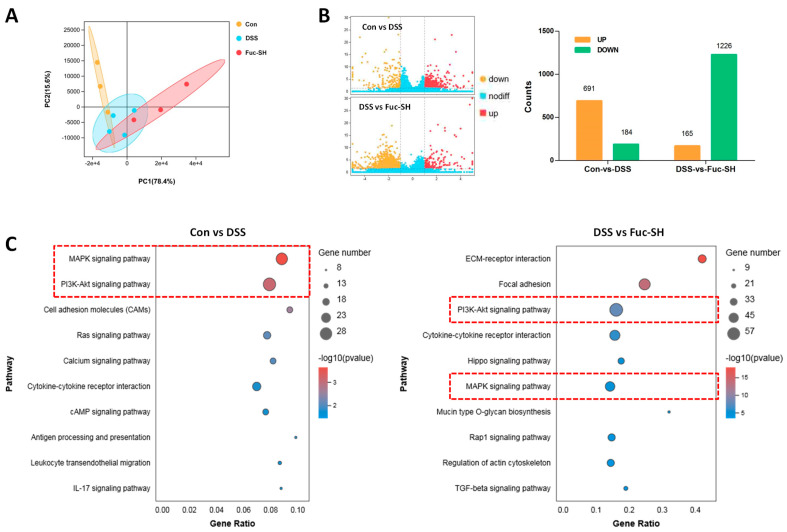
Colonic transcriptomics analysis of Fuc-S treatment of DSS-induced chronic colitis in mice: (**A**) PCA analysis of the Con, DSS, and Fuc-SH groups. (**B**) Comparative gene analysis—Con vs. DSS and DSS vs. Fuc-SH. (**C**) Top 10 KEGG pathways involved in intestinal inflammation in Con vs. DSS and DSS vs. Fuc-SH. Con: control, Fuc-SH: Fuc-S with high dosage (200 mg/kg).

**Figure 6:**
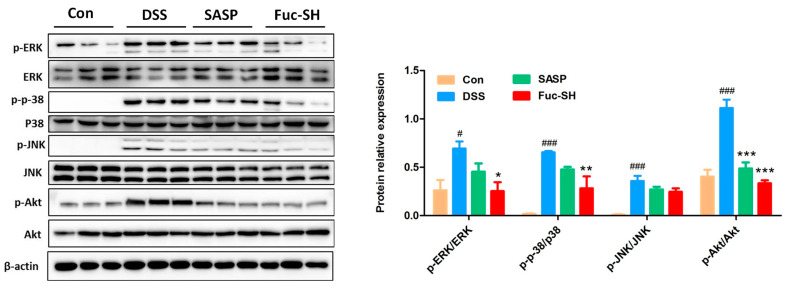
Fuc-S reduced the expression of p-Akt, p-ERK, p-JNK, and p-p38 MAPK in the colon tissues of mice with DSS-induced chronic colitis: (**A**) The expression of ERK, p-ERK, p-38, p-p38, JNK, p-JNK, Akt, p-Akt, and β-actin. (**B**) Relative intensities of p-ERK/ERK, p-p38/p-38, p-JNK/JNK, and p-Akt/Akt. Results are expressed as the mean ± SEM and were analyzed using ANOVA followed by Duncan’s multiple range test; # *p* < 0.05, and ### *p* < 0.001, compared to the Con group; * *p* < 0.05, ** *p* < 0.01, and *** *p* < 0.001, compared to the DSS group. Con: control, SASP: salazosulfadimidine, Fuc-SH: Fuc-S with high dosage (200 mg/kg).

In order to explore the possible mechanisms of Fuc-S in protecting against DSS-challenged chronic colitis in mice, the gene expression profiles of colon tissues were analyzed by transcriptomics. As shown in Figure 5A, the score plot from principal component analysis (PCA) showed good separations between the control group, model group, and Fuc-S treatment (200 mg/Kg) group. In parallel, comparative gene analysis showed that 691 and 184 genes in the colon tissue of DSS-induced colitic mice were drastically upregulated and downregulated, respectively, in comparison with those of the control mice. After Fuc-S treatment (200 mg/Kg), 165 and 1226 genes in the colon tissue of DSS-induced colitic mice were significantly upregulated and downregulated, respectively (Figure 5B). Afterwards, significant KEGG pathways involved in intestinal inflammation with *p*-values < 0.05 were screened out. The differentially expressed genes (DEGs) were mainly enriched in 10 associated pathways, including the “MAPK signaling pathway”, “PI3K-Akt signaling pathway”, “Cytokine-cytokine receptor interaction”, “Cell adhesion molecules”, “Ras signaling pathway”, “Rap1 signaling pathway”, and “Hippo signaling pathway”. Among these pathways, the “MAPK signaling pathway” and “PI3K-Akt signaling pathway” were the most prominent in both “Control-vs-DSS” and “DSS-vs.-Fuc-S” according to the enrichment gene numbers (Figure 5C). Subsequently, the regulatory effects of Fuc-S on MAPK and PI3K-Akt signaling in the colon tissues of colitic mice were verified using Western blots. As shown in Figure 6, the expression levels of p-Akt, p-ERK1/2, p-JNK, and p-p38 MAPK were significantly upregulated in the colon tissues of DSS-induced colitic mice, whereas these increases were dramatically downregulated after Fuc-S (200 mg/Kg) treatment. These findings indicate that the protective effects of Fuc-S against DSS-induced chronic colitis in mice are involved in regulating MAPK and PI3K-Akt signaling.

#### 2.2.4. Fuc-S Regulates the Intestinal Microecology of DSS-Induced Chronic Colitic Mice

**Figure 7:**
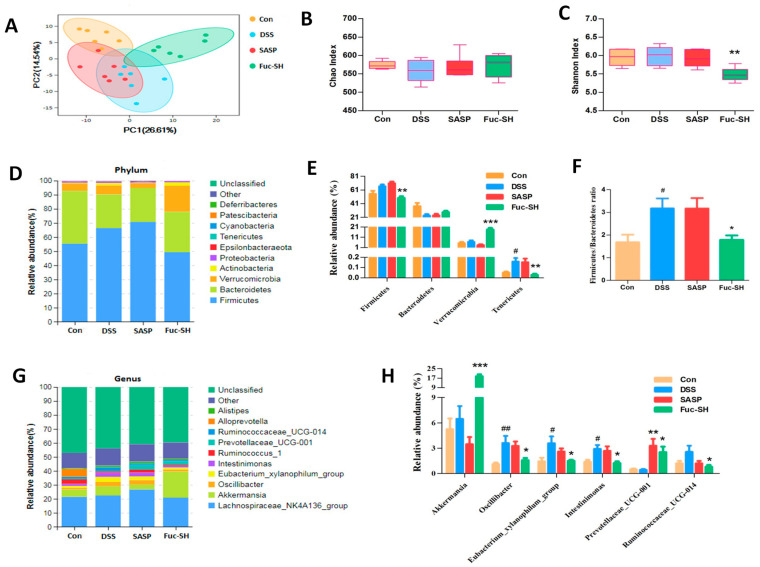
Fuc-S modulated the gut microbiota structure of DSS-induced chronic colitic mice: (**A**) PCA analysis of the Con, DSS, SASP, and Fuc-SH groups. (**B**) Effect of Fuc-S on the Chao index. (**C**) Effect of Fuc-S on the Shannon index. (**D**) Effect of Fuc-S on the gut microbiota structure at the phylum level. (**E**) Effect of Fuc-S on changes in principal bacteria at the phylum level. (**F**) Effect of Fuc-S on changes in the Firmicutes/Bacteroidetes ratio. (**G**) Effect of Fuc-S on the gut microbiota structure at the genus level. (**H**) Effect of Fuc-S on changes in principal bacteria at the genus level. Results are expressed as the mean ± SEM. (*n* = 6) and were analyzed using ANOVA followed by Duncan’s multiple range test; # *p* < 0.05, and ## *p* < 0.01, compared to the Con group; * *p* < 0.05, ** *p* < 0.01, and *** *p* < 0.001, compared to the DSS group. Con: control, SASP: salazosulfadimidine, Fuc-SH: Fuc-S with high dosage (200 mg/kg).

As the dysbiosis of intestinal microecology plays an important pathogenic role in the development of IBD [42], we also determined the effects of Fuc-S on the gut microbiota using 16S rRNA sequencing analysis. As shown in Figure 7A, the PCA score plot showed that the control group was clearly separated from the DSS model group along the first principal component (PC1)’s axis; however, the Fuc-S-treated group showed a clear separation from the DSS model group along the second principal component (PC2)’s axis, indicating that there was a good clustering trend among the samples in each group. In parallel, the bacterial diversity measured by the Chao diversity index showed no difference in the Fuc-S-treated group compared with the DSS model group (Figure 7B), but the diversity measured by the Shannon diversity index revealed that the value of the Fuc-S-treated group was significantly decreased (Figure 7C), suggesting that Fuc-S treatment could regulate the gut microbiota structure of chronic colitic mice. To assess specific changes in the gut microbiota, the relative abundances of the predominant taxa in these groups were compared. The intestinal microbial compositions at all taxonomic levels were significantly different. At the phylum level, the fecal microbiota microbial communities in all groups were Firmicutes, Bacteroidetes, and Verrucomicrobia (Figure 7D). Compared to the DSS model group, the relative abundance of Firmicutes was significantly downregulated, whereas the relative abundance of Verrucomicrobia was significantly upregulated after Fuc-S treatment (Figure 7E). Additionally, the increased ratio of Firmicutes/Bacteroidetes was dramatically reduced after Fuc-S treatment (Figure 7F). At the genus level, the fecal microbiota was occupied by the Lachnospiraceae_NK4A136_group, *Alloorevotella*, and *Akkermansia* (Figure 7G). Compared to the DSS model group, the relative abundances of *Akkermansia* and *Prevotellaceae*_UCG_001 in the fecal microbial communities of the Fuc-S treated group were significantly increased, while the relative abundances of the Eubacterium_xylanophilum_group, *Intestinimonas*, Ruminococcaceae_UCG-014, and *Oscillibacter* were observably decreased (Figure 7H). Taken together, these results showed that Fuc-S treatment could regulate the structure and abundance of the gut microbiota in chronic colitic mice.

#### 2.2.5. Fuc-S Modulates Host–Microbe Tryptophan Metabolism in DSS-Induced Chronic Colitis

Growing evidence suggests that microbiota dysbiosis could modulate the development of IBD in a tryptophan-metabolism-dependent manner [43,44,45,46,47]. We therefore quantified the metabolites of tryptophan in the feces and sera of chronic colitic mice using UPLC/Q-TOF-MS. As shown in Figure S7A, in serum samples, the metabolites kynurenic acid, kynurenine, serotonin, and tryptamine were significantly upregulated in DSS-treated mice, but these increases were diminished dramatically after Fuc-S treatment. Additionally, the metabolites indole-3-acetic acid, indole-3-carboxaldehyde, and indole-3-acetronitrile in the sera of DSS-treated mice were decreased, while Fuc-S treatment could significantly increase the concentration of indole-3-acetic acid in the serum. In parallel, the same regulatory effect of Fuc-S on tryptophan metabolism in feces was observed. The increases in the metabolites kynurenic acid, kynurenine, serotonin, and tryptamine in the feces of colitic mice were significantly suppressed, and the decreases in the metabolites indole-3-acetic acid, indole-3-carboxaldehyde, and indole-3-acetronitrile were greatly reversed, after Fuc-S treatment (Figure S7B). These results suggest that Fuc-S treatment could modulate host–microbe tryptophan metabolism in chronic colitic mice.

## 3. Discussion

In the present study, we suggested an ultrasonic method to degrade heterogeneous fucoidan into Fuc-S—a new homogeneous polysaccharide with a molecular weight of 156 kDa—and investigated its protective effects against DSS-induced chronic colitis in mice. Our data revealed that Fuc-S has a backbone consisting of →3)-α-L-Fucp-(1→, →4)-α-L-Fucp-(1→ and →3, 4)-α-D-Glcp-(1→. Oral administration of Fuc-S significantly attenuated the severity of DSS-induced chronic colitis, as evidenced by the improved clinical symptomatic features, reducing colon shortening, colon injury, colonic inflammatory cell infiltration, and colonic pro-inflammatory cytokine production. Moreover, Fuc-S significantly inhibited colonic phosphorylation of Akt, p-38, ERK, and JNK, increased the relative abundances of *Akkermansia* and Prevotellaceae UCG 001, and reduced the relative abundances of the *Eubacterium xylanophilum* group, *Intestinimonas*, Ruminococcaceae UCG-014, and Oscillibacter in the gut, as well as decreasing the concentrations of kynurenine, serotonin, and tryptamine and increasing the concentrations of indole-3-acetic acid in the sera and feces of colitic mice.

Fucoidans are sulfated homo- and heteropolysaccharides, and their structures are highly variable depending on algal species, age, geographic origin, and season of harvesting [48]. The most widely accepted structural models consist of a linear or branched sulfated L-fucopyranoside backbone linked only by alternating α-(1→3) and α-(1→4) linkages and α-(1→4) and α-(1→3) linkages [49,50]. In addition, some other structural models can also be found in different algal species. For example, Usoltseva et al. found that the backbone of fucoidans produced by *Laminaria cichorioides* and *Laminaria longipes* contained α-(1→2)-linked fucopyranoside residues [38]. Koh et al. found that fucoidan produced by *Undaria pinnatifida* contained an alternating fucose–galactose backbone linked together via 1,3-glycosidic bonds with sulfation at the C2 and C4 positions [51]. Moreover, Ermakova et al. found that fucoidan from *Ecklonia cava* contained a fucose–glucose backbone [52]. In addition, we found that Fuc-S—a degraded polysaccharide produced from *Fucus vesiculosus*—also has a fucose–glucose backbone. This finding further confirmed the presence of glucose in the backbone of fucoidan.

As colonic inflammation induced by repeated DSS is similar to the phenotypic features of human IBD, a murine model of DSS-induced chronic colitis was selected. Using this murine model, we found that Fuc-S significantly attenuated the clinical symptomatic features and colon injury of DSS-induced chronic colitic mice, including body weight loss, disease activity index, colon shortening, and colon tissue damage, and Fuc-S (200 mg/kg) exerted better protective effects in comparison to SASP treatment. Cells such as neutrophils, monocytes, and macrophages were increased in the colon and secreted various inflammatory mediators, playing important roles in the development of DSS-induced colitis. Our data revealed that Fuc-S is effective in reversing colonic inflammatory cell infiltration, as monitored by CD11b expression. Additionally, Fuc-S also successfully prevented colitis by inhibiting the production of TNF-α, IFN-γ, IL-6, and IL-17A in the colons of colitic mice. Thus, Fuc-S might be a promising candidate for the treatment of colitis.

MAPKs are the main signal transduction pathways involved in regulating the inflammatory and immune responses, including three representative pathways—namely, the p38/MAPK, ERK, and JNK pathways. Previous studies have demonstrated that the activities of MAPKs are profoundly increased when intestinal epithelial injury occurs, and that chemical inhibition of MAPK signaling pathways is effective in suppressing the colonic inflammation status of experimental colitic animals [53,54,55]. Additionally, accumulating evidence also suggests that the PI3K/AKT signaling pathway is also involved in the pathogenesis of IBD, and inhibitors of PI3K/AKT have been proven to alleviate colonic inflammation [56,57,58]. Based on the RNA-Seq analysis, the Fuc-S-treated group exhibited differential expression of a specific set of genes associated with PI3K/AKT and MAPK signaling compared to the DSS model group. Subsequently, the results of Western blots further verified that those upregulated p-Akt, p-ERK1/2, p-JNK, and p-p38 MAPK proteins in the colon tissues of colitic mice were significantly suppressed by treatment with Fuc-S. These results suggest that the anti-inflammatory activity of Fuc-S may be at least partially mediated by inactivation of PI3K/AKT and MAPK signaling.

The *Firmicutes/Bacteroidetes* ratio is widely accepted to have an important influence in maintaining normal intestinal homeostasis. Compared to the mucosal microbiota communities of healthy controls, CD patients had a significantly higher proportion of Bacteroidetes, and both UC and CD biopsies showed slightly decreased abundance of *Firmicutes* [59,60]. In the present study, it was clear that the *Firmicutes/Bacteroidetes* ratio was sharply increased in the colons of DSS-induced colitic mice, while this increase was rescued after Fuc-S treatment, indicating that Fuc-S treatment is favorable for maintaining intestinal homeostasis. Recently, several studies have shown that the bacterium *Akkermansia* was highly associated with healthy mucosa in the context of multiple disorders [61,62,63], and the abundance of *Akkermansia* was markedly reduced in IBD patients [64]. Oral administration of *Akkermansia* ameliorated the progression of colitis [64]. Our data also showed that Fuc-S treatment increased the relative abundance of *Akkermansia* in the colons of DSS-induced colitic mice. Moreover, *Ruminococcaceae* species are positively associated with the severity of colonic inflammation [65,66,67]. In this study, the relative abundance of Ruminococcaceae_UCG-014 was significantly decreased after Fuc-S treatment. These findings indicate that Fuc-S treatment could regulate gut microbiota dysbiosis in chronic colitic mice.

Intestinal homeostasis is co-governed by the host and the gut microbiota, and metabolites from both are key actors. Alterations in the composition and content of metabolites such as tryptophan are related to the pathogenesis of IBD [68]. The free tryptophan in the body is metabolized through three pathways (kynurenine, serotonin, and indole pathways). Among them, the kynurenine and serotonin pathways are predominantly host pathways [68,69]. Several studies have shown that increased tryptophan metabolism through the kynurenine and serotonin pathways to produce high levels of kynurenine, serotonin, and tryptamine is associated with the activity of IBD [44,47]. In this study, the concentrations of kynurenine, serotonin, and tryptamine in the sera and feces of colitic mice were all significantly decreased after Fuc-S treatment. On the other hand, the indole pathway is controlled by the gut microbiota. The free tryptophan in the gut can be metabolized by the microbiota into a range of indole metabolites (including indole-3-acetic acid, indole-3-carboxaldehyde, indole-3-acetronitrile, indole-3-acetaldehyde, indoleacrylic acid, etc.) [68]. These indole metabolites are agonists of the aryl hydrocarbon receptor (AhR), with potential anti-inflammatory properties against colitis [43,44]. Our data showed that the decreased levels of the indole metabolite indole-3-acetic acid in the sera and feces of colitic mice were greatly reversed after Fuc-S treatment. Collectively, these results clearly indicate that Fuc-S treatment could modulate host–microbe tryptophan metabolism in chronic colitic mice.

However, there are still several issues that need to be clarified—for example, whether the anti-inflammatory effect of Fuc-S is better than that of fucoidan from *Fucus vesiculosus*, about the nature of its anti-inflammatory properties and underlying mechanisms in vitro, and how Fuc-S regulates tryptophan metabolism. Further investigations are warranted to better understand the protective effects and mechanisms of Fuc-S underlying the present observations.

## 4. Materials and Methods

### 4.1. Preparation of Fuc-S

Fucoidan, originating from *Fucus vesiculosus*, was attained from Shanghai Eysin Biotechnology Co., Ltd. (Shanghai, China), and it was prepared using water extraction (liquid-to-material ratio of 10:1 (mL/g); reflux extraction: 2 h) and alcohol precipitation (alcohol-to-sample ratio of 4:1 (mL/g)) methods (purity ≥ 85%, phenol–sulfuric acid method) [70]. The brown alga *Fucus vesiculosus* was bought from Shanghai Eysin Biotechnology Co., Ltd. (Shanghai, China). The brown alga *Fucus vesiculosus* was identified by Professor Qiancheng Zhao from Dalian Ocean University. Fuc-S was prepared by the degradation of fucoidan using ultrasonication in a Scientz-IID ultrasonic cell disrupter (Ningbo Scientz Biotechnology, Ningbo, China). In brief, a 1 g/L aqueous solution of fucoidan was prepared and degraded by ultrasound at a 950 W maximum output power and 25 kHz frequency under stirring for 5 h in an ice-water bath. The Fuc-S solution was then freeze-dried and stored for further study.

### 4.2. Molecular Weight Determination

The Mw of Fuc-S was determined by high-performance size-exclusion chromatography (HPSEC) equipped with a multi-angle laser light scattering (MALLS) detector (DAWN HELEOS Ⅱ, Wyatt Technology Co., Santa Barbara, CA, USA) and an Optilab T-rEX differential refractive index detector (RID) in a Waters 2695 HPLC system (Wyatt technology, Santa Barbara, CA, USA). Several columns were used, including a guard column, Shodex OHpak SB-G (50 mm × 6 mm, Shodex China Co., Ltd., Shanghai, China), and Shodex OHpak SB-806 HQ (300 mm × 8 mm, Shodex China Co., Ltd., Shanghai, China). An aqueous solution of NaNO_3_ (0.1 M) was prepared as the mobile phase. The Mw was calculated using Astra software (Version 6.0.2, Wyatt Tech. Corp., Santa Barbara, CA, USA).

### 4.3. Monosaccharide Composition Determination

The method of pre-column derivatization HPLC with 1-phenyl-3-methyl-5-pyrazolone (PMP) was used to measure the monosaccharide composition, as previously described in [71]. In brief, Fuc-S was first hydrolyzed by trifluoroacetic acid (TFA, 4M) at 120 °C for 2 h and, subsequently, derivatized by the addition of a 0.3 M NaOH solution and 0.5 M PMP methanol solution. After neutralization with 0.3 M hydrochloric acid, the solution was extracted with chloroform three times. The extract was analyzed using an Agilent 1200 Infinity HPLC System with a refractive index detector. A Shiseido C18 column (5 μm, 4.6 mm × 250 mm; Shiseido, Ginza, Tokyo, Japan) was used and maintained at 25 °C. The mobile phases were eluent A (0.1M KH_2_PO_4_ solution (pH 6.8)) and eluent B (acetonitrile), running at a flow rate of 1 mL × min^−1^. The injection volume was 10 μL.

### 4.4. Methylation Analysis

Fuc-S was methylated as described in previously published papers, with minor modifications [70,71,72]. In brief, Fuc-S was first subjected to carboxyl reduction with NaBH_4_. After adjusting the pH to acidic, the sample was freeze-dried. Subsequently, the polysaccharide was methylated with NaOH-DMSO and methyl iodide. The reaction mixture was first extracted with chloroform and then hydrolyzed with trifluoroacetic acid. Subsequently, the hydrolysates were dissolved in 1% (w/w) NaOH, and then NaBH_4_ and glacial acetic acid were successively added. Next, the sample was dried under nitrogen and then acetylated with acetic anhydride and pyridine. The acetylated products were extracted with chloroform and analyzed using a gas chromatograph–mass spectrometer (GC-MS) (Agilent 7890B-7000D, Agilent, Santa Clara, CA, USA). An Econo-CapTM EC-1 column (30 m × 0.32 mm × 0.25 mm; Alltech, Chicago, IL, USA) was used and operated at 70 eV with helium at a flow rate of 0.8 mL/min, under the following heating program: the GC oven temperature was set to 140 °C and held for 5 min, before increasing by 8 °C/min to 220 °C and holding for 30 min, and then increasing by 10 °C/min to 240 °C and holding for 10 min.

### 4.5. FTIR and NMR Spectroscopy

The Fourier-transform infrared (FTIR) spectrum (400 × 4000 cm^−1^) of Fuc-S was analyzed using an FTIR spectrometer (Bruker Corporation, Karlsruhe, Germany) with the method of potassium bromide (KBr). The 1D and 2D nuclear magnetic resonance (NMR) spectra of Fuc-S were measured using a Bruker Avance III 600 M spectrometer (Bruker Corporation, Karlsruhe, Germany) at 298 K. Tetramethoxysilane (TMS) was used as an internal standard.

### 4.6. Animals and Experimental Design

Male C57BL/6 mice (Beijing Vital River Laboratory Animal Technology Co., Ltd., Beijing, China) aged 6–8 weeks (18–22 g) were acclimated for one week before the experiments. The mice were kept in an environment without specific pathogens at the animal house of Shenzhen University and were fed with standard mouse feed (21.6% protein, 3.7% fat, 61.6% sugar, 45% cellulose, 1.3% calcium, and 1.5% phosphorus.) and water ad libitum. All animal experiments were approved by the Animal Care Ethics Committee of Shenzhen University (No. 000002110144).

Chronic colitis was induced in the mice as described in our previous study, with slight modifications [39]. Briefly, five groups—namely, control, DSS model, DSS plus salazosulfadimidine (SASP—a positive reference agent; Sigma-Aldrich, St. Louis, MO, USA), DSS plus Fuc-S with high dosage, and DSS plus Fuc-S with low dosage—were set. All mice in the DSS-treated groups received three cycles of 1.8% DSS (MP Biomedicals, Ohio, USA) for 5 days and then water for 7 days to induce colitis (As shown in Figure 3A). From day 0, the mice were treated daily with Fuc-S, SASP, or water. The dosages of Fuc-S were set to 200 mg/kg and 100 mg/kg according to the preliminary experiment. In parallel, the dosage of SASP was set to 200 mg/kg according to the literature [6]. The mice’s body weight and disease activity index (DAI) were measured and recorded every day. The DAI was determined by the percentage of weight loss, degree of stool consistency, and stool bleeding, as described in our previous study [6].

### 4.7. Histopathological Evaluation and Immunofluorescence (IF) Staining

At the end of the study, the mice were euthanatized, and the length of the colon was determined. Subsequently, the colon sections were fixed in 4% paraformaldehyde for 24 h and then embedded in paraffin, stained with hematoxylin–eosin (HE) (Sigma-Aldrich, St, Louis, MO, USA), and then evaluated and scored in a blind manner as described in our previous study [40]. For analysis of CD11b-positive inflammatory infiltration, the colon sections were first incubated with hydrogen peroxide (3%). Subsequently, the sections were incubated overnight with monoclonal murine anti-CD11b (Invitrogen) at a dilution of 1:200 at 4 °C, and subsequently with Alexa Fluor^®^ 488 goat anti-mouse IgG at a dilution of 1:500 (Invitrogen) and 4,6-diamidino-2-phenylindole (DAPI) (Sigma-Aldrich) in the dark. After washing in water three times, the FITC and DAPI images were examined under an immunofluorescence microscope (Olympus, Tokyo, Japan) with a 20 × objective lens.

### 4.8. Luminex Detection and Western Blot Analysis

The colon tissue was homogenized with PBS, and the levels of IL-6, TNF-α, IL-1β, and IL-17A in the colon tissues were determined using a Luminex ELISA kit (R&D systems, Minneapolis, MN, USA) according to the manufacturer’s instructions. Additionally, Western blot analysis was performed as described in our previous study [6]. The antibodies used were as follows: primary antibodies against Akt, p-Akt, p38, p-p38, ERK, p-ERK, JNK, p-JNK, and β-actin (Cell Signaling, MA, USA). The fluorescence signals were visualized with ECL reagents and quantified using ImageJ software.

### 4.9. Transcriptomics by RNA-Sequencing Analysis

The total RNA from about 50 mg of colon tissues was extracted using TRIzol reagent, and then the purity, concentration, and integrity of the total RNA were analyzed using NanoDrop and BioAnalyzer II reagents (Agilent, CA, USA). Next, the RNA-Seq library was established for mRNA sequencing. The sequencing was conducted using an Illumina HiSeq 4000 at the LC-BIO (Hangzhou, China). Gene expression levels were assessed by fragments per kilobase of transcript per million fragments mapped (FPKM). The differentially expressed genes (DEGs) were defined by fold change > 2 and raw data *p* < 0.05. Database functionality annotations for DEGs were performed by Gene Ontology (GO) analysis and Kyoto Encyclopedia of Genes and Genomes (KEGG) enrichment analysis.

### 4.10. Microbiota Analysis

The fecal 16S rRNA analysis was performed as previously described [73]. In brief, total microbial DNA from feces was extracted using the QIAamp DNA Micro Kit (TIANGEN, China). The V3-V4 regions of the 16S rDNA gene were amplified using the forward primer 338F (ACT CCT ACG GGA GGC AGC AG) and the reverse primer 806R (GGA CTA CHV GGG TWT CTA AT) [74,75,76].

After PCR reactions, purification, and quantification, the PCR products were sequenced by Allwegene using the Illumina MiSeq PE300 sequencing platform (Illumina Inc., San Diego, CA, USA). Briefly, the raw fast files were filtered using Cutadapt (V1.9.1). The OTUs were clustered with a cutoff value of 97% similarity using UPARSE (version 7.0.1001), and chimeric sequences were removed using UCHIME. The community composition analysis was performed and classified using the RDP classifier (http://rdp.cme.msu.edu/) based on the SILVA ribosomal RNA gene database [77,78,79].

### 4.11. Metabolic Analysis of Tryptophan Metabolism

The analysis of tryptophan metabolism in serum and feces was performed using an Agilent ultra-performance liquid chromatography (UPLC) system coupled with a triple-quadrupole (QQQ) MS6460 mass spectrometer. In brief, serum (100 μL) was extracted with methanol (400 μL) and vigorously vortexed, and then centrifuged at 14,000 rpm for 15 min at 4 °C. In parallel, approximately 100 mg of feces of the mice was homogenized with 10-fold 80% methanol (m/v) and centrifuged under the same conditions. Approximately 200 μL of the upper supernatants of serum and fecal extracts was transferred to new tubes for LC-MS analysis. A Waters BEH C18 column (2.1 mm × 50 mm, 1.7 μm) was used and maintained at 35 °C. The mobile phases were eluent A (0.1% formic acid in water, *v/v*) and eluent B (0.1% formic acid in acetonitrile, *v/v*), running a liner gradient program at a flow rate of 1mL × min^−1^ as follows: 2% B from 0 to 0.5 min, 2–3% B from 0.5 to 4 min, 30–100% B from 4 to 6 min, 100% B from 6 to 8 min, 100–2%B from 8 to 8.1 min, and maintaining 2% B from 8.1 to 10 min. The MS analysis was performed with a quadrupole time-of-flight (Q-TOF) tandem mass spectrometer (Agilent Technologies, Santa Clara, CA, USA) equipped with electrospray ionization (ESI) in negative ionization mode, as described in our previous study [6]. The standards, MRM transitions, and retention times are listed in Table S1 that can be found in Supplementary Material Section.

### 4.12. Statistical Analysis

GraphPad Prism 5.0 software (GraphPad Software Inc., San Diego, CA, USA) was used to analyze the data. The results are expressed as the mean ± standard error of the mean (SEM). Differences among three or more groups were evaluated using one-way ANOVA, followed by Duncan’s multiple range test. Probabilities of <0.05 were considered statistically significant.

## 5. Conclusions

In the present study, a new homogeneous ultrasonic degraded polysaccharide of Fuc-S with a molecular weight of 156 kDa was prepared from fucoidan produced by *Fucus vesiculosus*. The backbone of this polysaccharide consisted of →3)-α-L-Fucp-(1→, →4)-α-L-Fucp-(1→ and →3, 4)-α-D-Glcp-(1→, rarely observed in other fucoidans. For conformation analysis, Fuc-S was found to have a random coil conformation in 0.9% NaCl solution, and in vivo assays confirmed that Fuc-S could effectively alleviate DSS-induced chronic colitis in mice. This favorable effect was associated with the suppression of inflammatory cell infiltration and pro-inflammatory cytokine production via inhibition of PI3K/AKT and MAPK activation, as well as inhibiting host–microbe tryptophan metabolism and regulating the intestinal microbiota. It can be concluded that Fuc-S is a promising nutraceutical candidate for the treatment of IBD.

## Figures and Tables

**Table 1:** Methylation analysis of Fuc-S.

Retention Time (min)	Permethylated Alditol Acetate	Linkages	Molar Ratio	Mass Fragments (m/z)
12.975	2,4-Me_2_-Fuc	→3)-Fuc-(1→	0.43	263, 233, 201, 173, 155, 129, 111, 101, 87, 71, 57, 43
11.439	2,3-Me_2_-Fuc	→4)-Fuc-(1→	0.15	283, 233, 204, 162, 142, 131, 118, 99, 87, 71, 43
15.868	2,6-Me_2_-Glc	→3, 4)-Glc-(1→	0.11	305, 253, 231, 203, 185, 143, 129, 118, 87, 71, 43
13.449	3-Me-Glc	→2, 4, 6)-Glc-(1→	0.10	261, 231, 201, 142, 127, 118, 99, 85, 71, 43
14.393	2-Me-Gal	→3, 4, 6)-Gal-(1→	0.07	333, 267, 202, 183, 160, 139, 127, 118, 85, 57, 43
13.817	2,3,4-Me_3_-Gal	→6)-Gal-(1→	0.06	292, 249, 233, 203, 189, 173, 162, 129, 102, 87, 71, 43
15.254	2,4,6-Me_3_-Man	→3)-Man-(1→	0.03	277, 183, 162, 146, 131, 118, 84, 59, 43
17.324	2,3,6-Me_3_-Glc	→4)-GlcUA-(1→	0.02	277, 233, 187, 162, 142, 131, 118, 99, 87, 71, 43
18.548	2,4-Me_2_-Gal	→3, 6)-GalUA-(1→	0.01	305, 234, 189, 160, 139, 129, 118, 99, 87, 71, 43
11.002	3,4-Me_2_-Xyl	→2)-Xyl-(1→	0.01	191, 175, 162, 118, 99, 87, 71, 59, 43
12.214	3,4- Me_2_-Rha	→2)-Rha-(1→	0.02	234, 190, 159, 129, 111, 99, 71, 43

**Table 2:** ^1^H and ^13^C NMR data of Fuc-S.

Structure No.	Glycosyl Residues	Chemical Shift (ppm)							
		H1/C1	H2/C2	H3/C3	H4/C4	H5/C5	H6/C6	C=O	CH_3_
**A**	→3)-α-L-Fuc*p*-(1→	5.03/98.27	3.78/68.94	3.94/76.06	4.36/79.66	4.08/66.68	1.18/15.59		
**B**	→4)-α-L-Fuc*p*-(1→	5.22/99.79	3.72/68.39	3.89/73.06	3.79/77.75	4.02/80.02	1.24/19.71		
**C**	→3,4)-α-D-Glc*p*-(1→	5.15/91.89	3.50/71.45	3.89/76.29	4.10/77.35	3.58/70.06	3.76/60.31		
**D**	→6)-α-D-Glap-(1→	5.32/99.62	3.54/71.46	3.91/72.62	3.77/71.26	3.59/72.62	3.30/69.24	-/179.2	1.88/22.57
**E**	→2,4,6)-α-D-Glcp-(1→	4.96/100.32	3.88/77.34	4.50/80.70	3.58/76.75	3.75/71.21	3.32/69.24		
**F**	→3,4,6)-β-D-Glap-(1→	4.57/95.88	3.20/73.92	3.69/76.13	3.80/76.82	3.58/72.74	3.51/71.50	-/180.10	2.42/32.41

## Data Availability

Not applicable.

## Supplementary Materials

The following are available online at www.mdpi.com/article/10.3390/md21010016/s1, Figure S1: The dependence of radius of gyration on malor mass, Figure S2: Monosaccharide composition determination of Fuc-S, Figure S3: Enlarged ^1^H-^1^H COSY of Fuc-S, Figure S4: Enlarged ^1^H-^1^H TOCSY of Fuc-S, Figure S5: Enlarged ^1^H-^1^H ROESY of Fuc-S, Figure S6: Enlarged ^1^H-^1^H ROESY of Fuc-S, Figure S7: Fuc-S modulated host-microbe tryptophan metabolism of DSS-induced chronic colitis in mice, Figure S8: Predicted structure of Fuc-S, Table S1: The MRM transition for quantification tryptophan metabolites.
