# Supplementary material for: Fuc-S—A New Ultrasonic Degraded Sulfated α-l-Fucooligosaccharide—Alleviates DSS-Inflicted Colitis through Reshaping Gut Microbiota and Modulating Host–Microbe Tryptophan Metabolism
Source: Mar Drugs. 2022 Dec 25;21(1):16. doi: 10.3390/md21010016 (PMC9863236; doi:10.3390/md21010016)
Supplement: Supplementary file 1 [file marinedrugs-21-00016-s001.zip › marinedrugs-2059433-supplementary.pdf]

Structural characterization and anti-colitic effects of a new degraded polysaccharide from fucoidan

Haitao Xiao[a,1]*, Jinxiu Feng[b,1], Jiao Peng[c], Peigen Wu[a, d], Yaoyao Chang[a], Xianqian Li[a], Jinhui Wu[b], Haifeng Huang[a], Huan Deng[a], Miao Qiu[a], Yuedong Yang[b]*, Bin Du[b]*

[a]School of Pharmaceutical Sciences, Health Science Center, Shenzhen University, Shenzhen, China.
[b]Hebei Key Laboratory of Natural Products Activity Components and Function, Hebei Normal University of Science and Technology, Qinhuangdao, China.
[c]Department of Pharmacy, Peking University Shenzhen Hospital, Shenzhen, China.
[d]School of Pharmaceutical Sciences, Guizhou Medical University, Guizhou, China.

[1]These authors contributed equally to this work.
*Correspondence: Bin Du, Professor, Hebei Key Laboratory of Natural Products Activity Components and Function, Hebei Normal University of Science and Technology, Qinhuangdao, China. Tel: +86-335-8387040, fax: +86-335-8387040, E-mail: bindufood@aliyun.com; or Yuedong Yang, Professor, Hebei Key Laboratory of Natural Products Activity Components and Function, Hebei Normal University of Science and Technology, Qinhuangdao, China. Tel: +86 335 8387040, fax: +86 335 8387040, E-mail: kycyyd@126.com; Hai-tao Xiao, Ph.D. School of Pharmaceutical Sciences, Health Science Center, Shenzhen University, 518060, Shenzhen, China. Email: xhaitao@szu.edu.cn.

1. Supplementary Figures

1.1 Fig S1

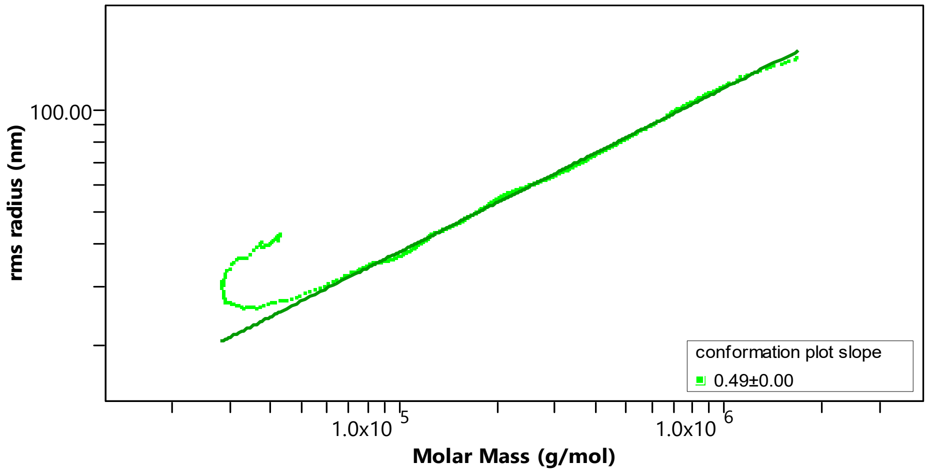

Supplementary Fig S1. The dependence of radius of gyration on malor mass.

Fig S1. showed the relationship between molecular weights and radius of gyration. Based on the theory of polymer solution, the slope of Rg-Mw value 0.33, 0.5-0.6 and 1 showed chain shapes of sphere, random coil, and rigid rod, respectively (Meng et al., 2018; Nie et al., 2020). As calculated from Fig. S1., the slope of Rg-Mw was 0.49, which indicated that fucoidan existed as a random coil in aqueous solution. The radius of gyration has applications in structural, mechanical and molecular engineering. It is defined as the distance between an axis and the point of maximum inertia in a rotating system.

1.2 Fig S2

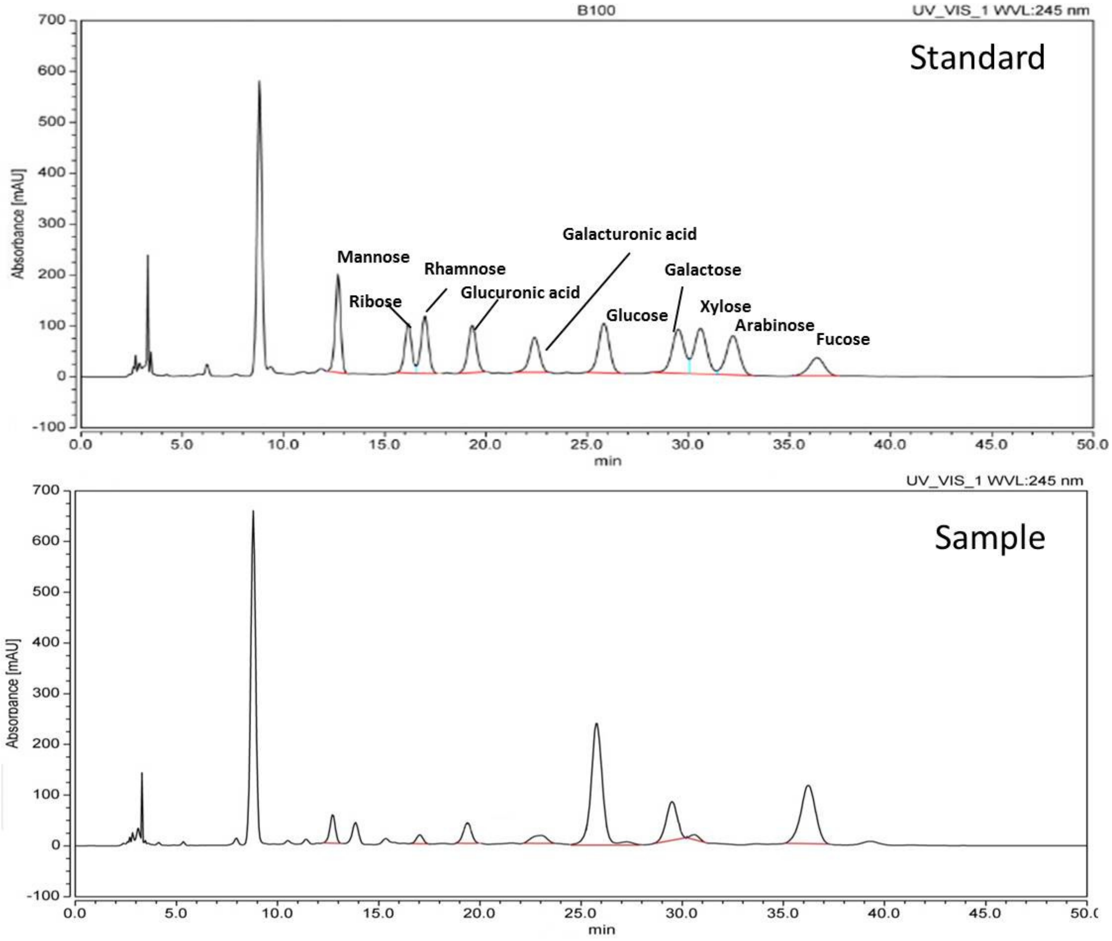

Fig S2 Monosaccharide composition determination of **Fuc-S**.

1.3 Fig S3

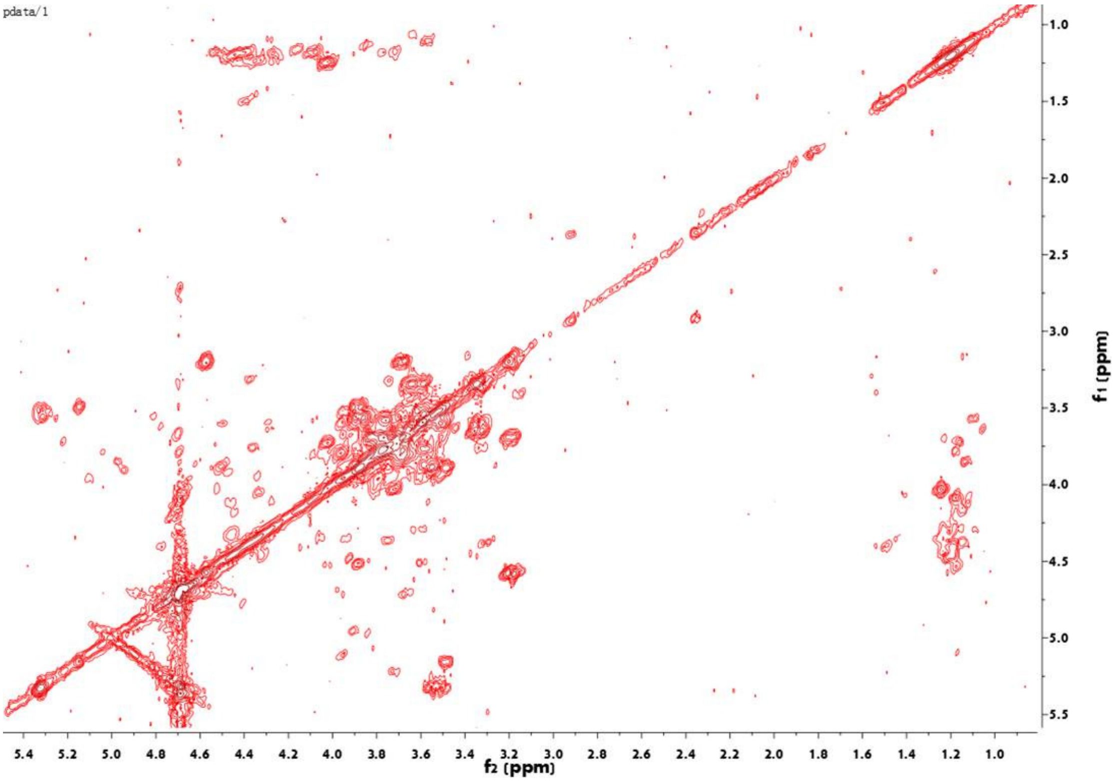
pdata/1
f1 (ppm)
f2 (ppm)

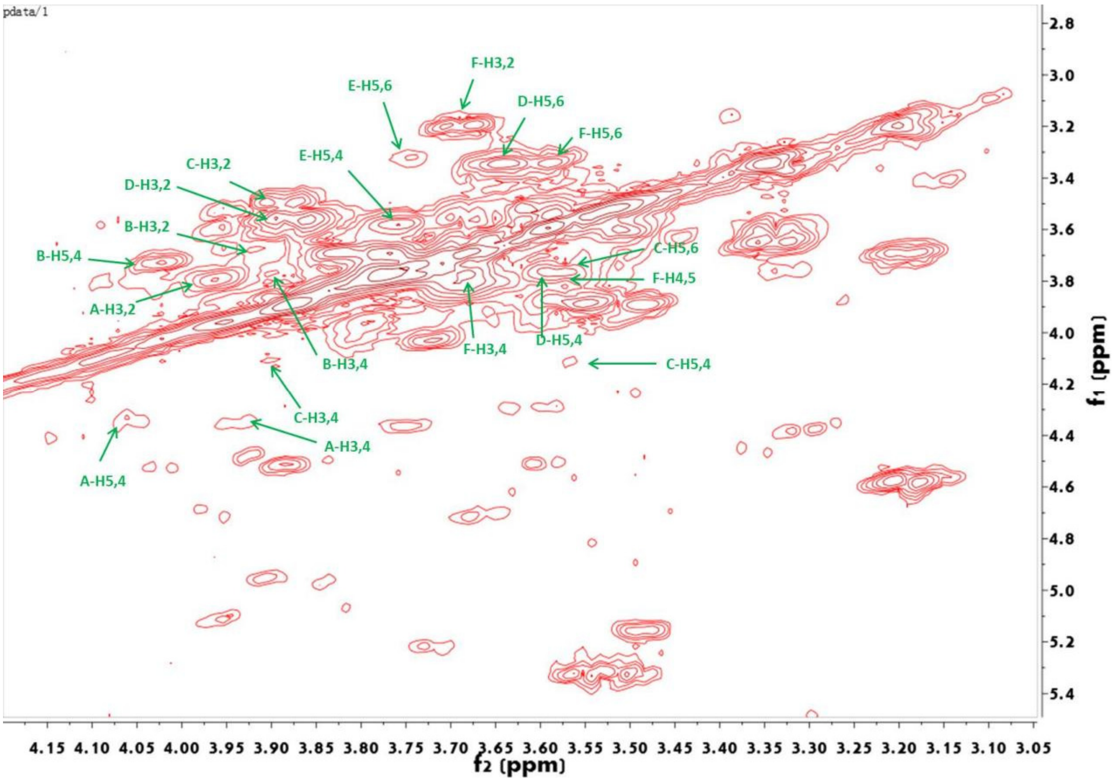
pdata/1
F-H3,2
E-H5,6
D-H5,6
F-H5,6
E-H5,4
C-H3,2
D-H3,2
B-H3,2
B-H5,4
A-H3,2
C-H5,6
F-H4,5
D-H5,4
B-H3,4
F-H3,4
C-H5,4
C-H3,4
A-H3,4
A-H5,4
f1 (ppm)
f2 (ppm)

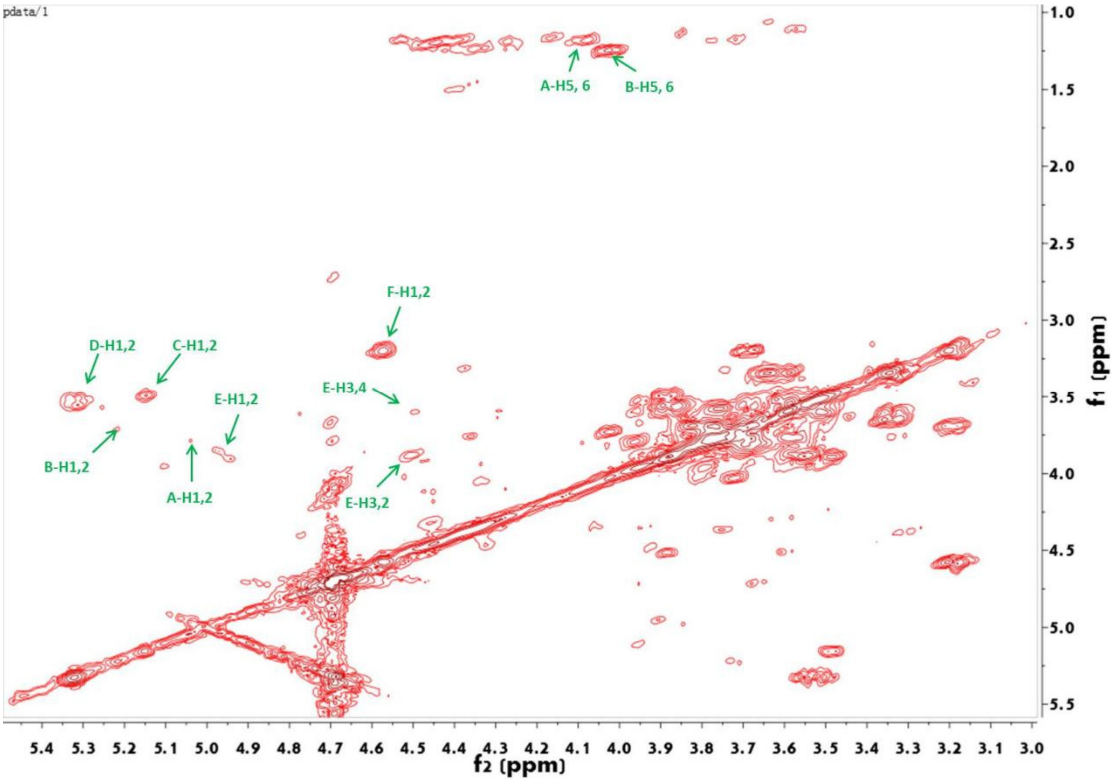

Fig S3 Enlarged ^1H-^1H COSY of Fuc-S

1.4 Fig S4

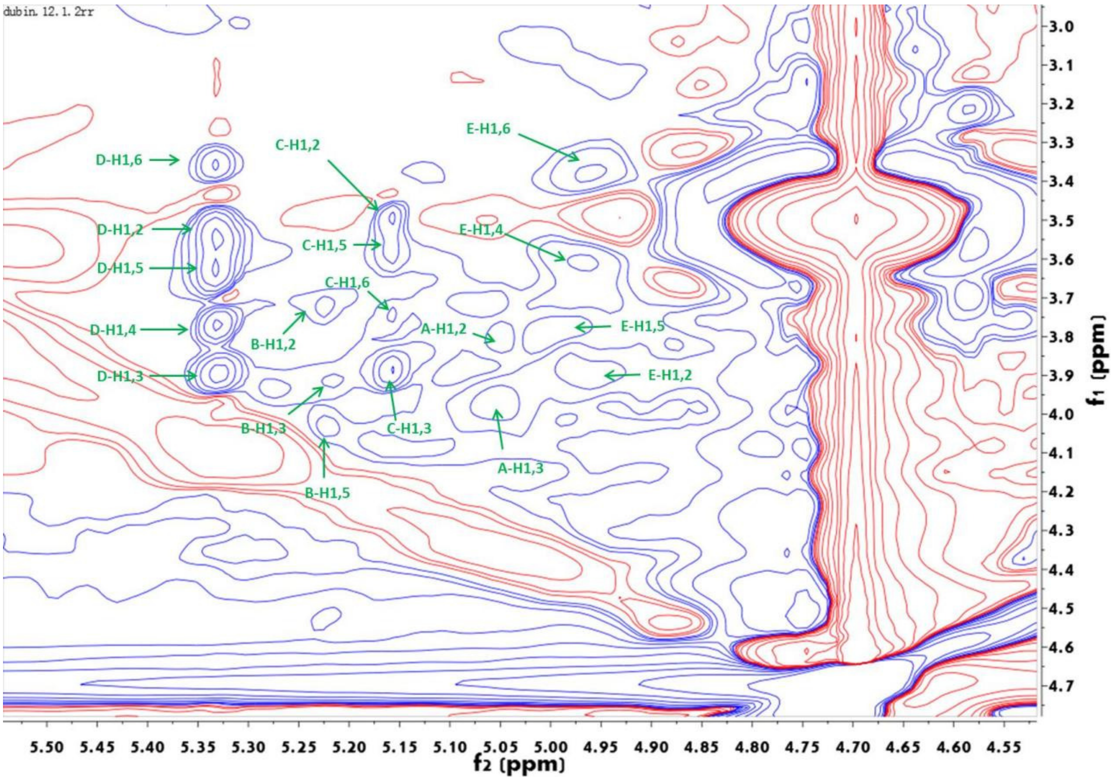

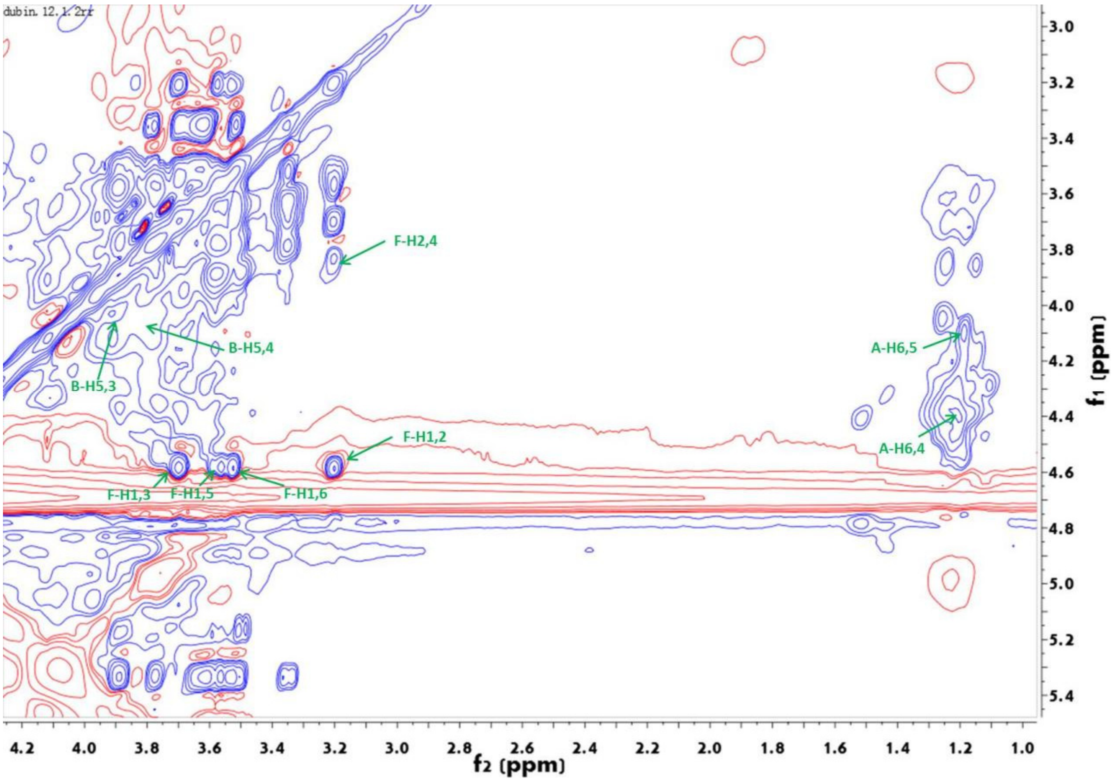

Fig S4 Enlarged ^{1}H-^{1}H TOCSY of Fuc-S.

1.5 Fig S5

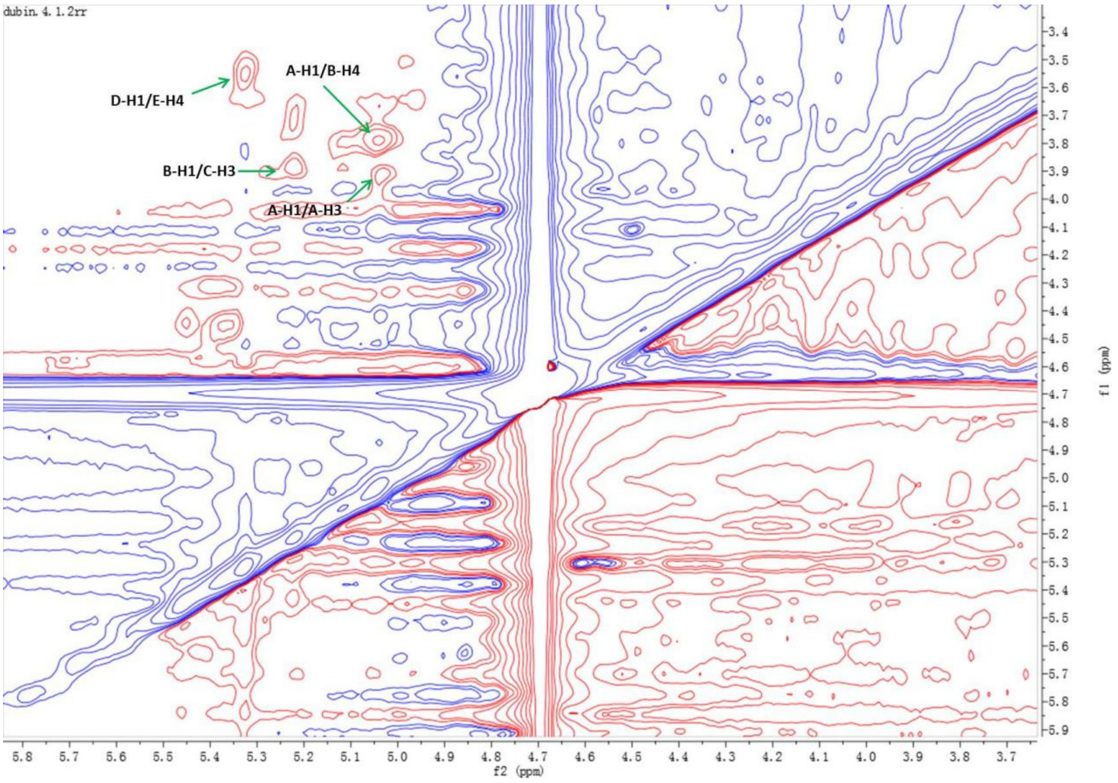

Fig S5 Enlarged ^{1}H-^{1}H ROESY of Fuc-S.

1.6 Fig S6

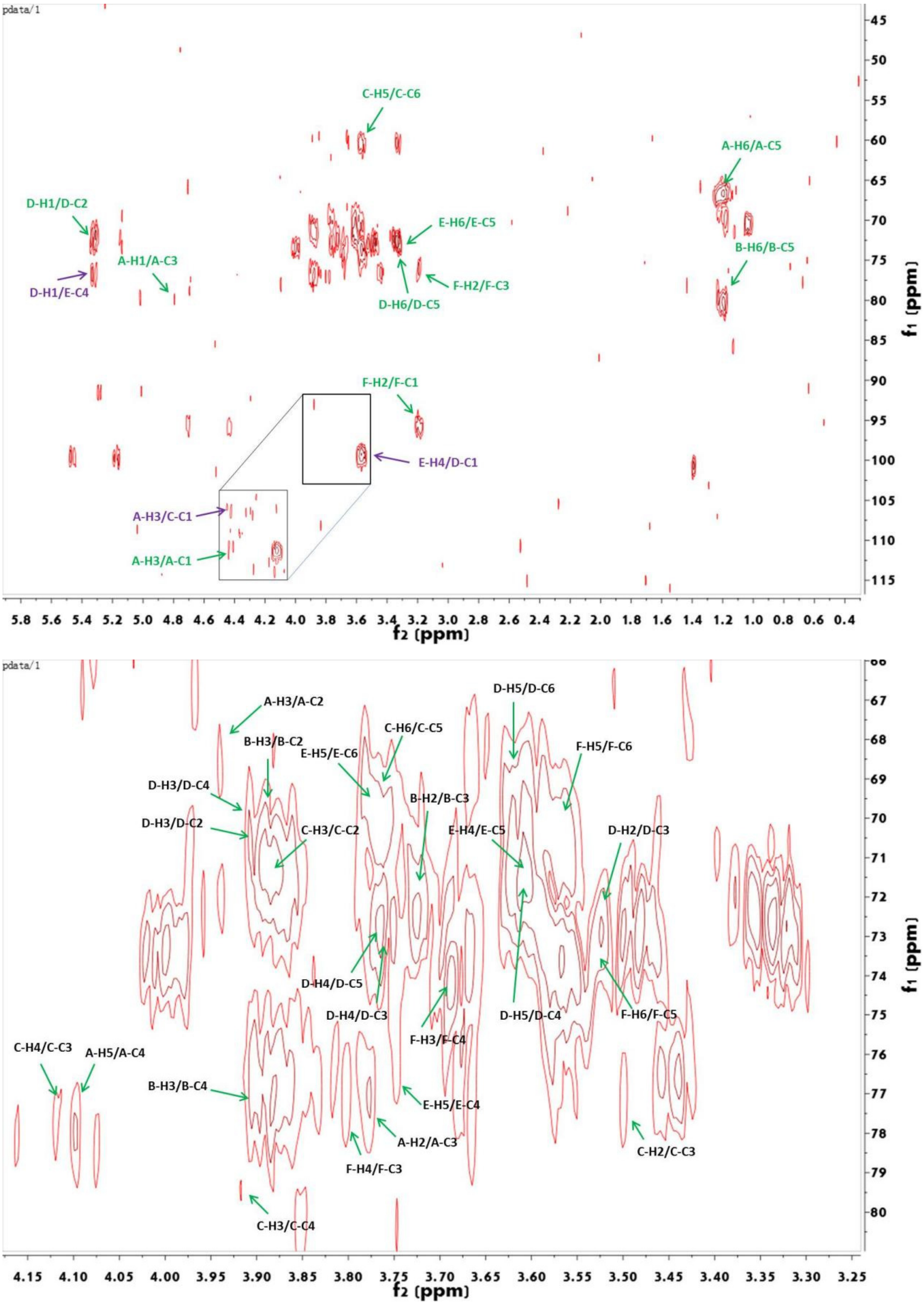

Fig S6 Enlarged ^{1}H-^{1}H ROESY of Fuc-S.

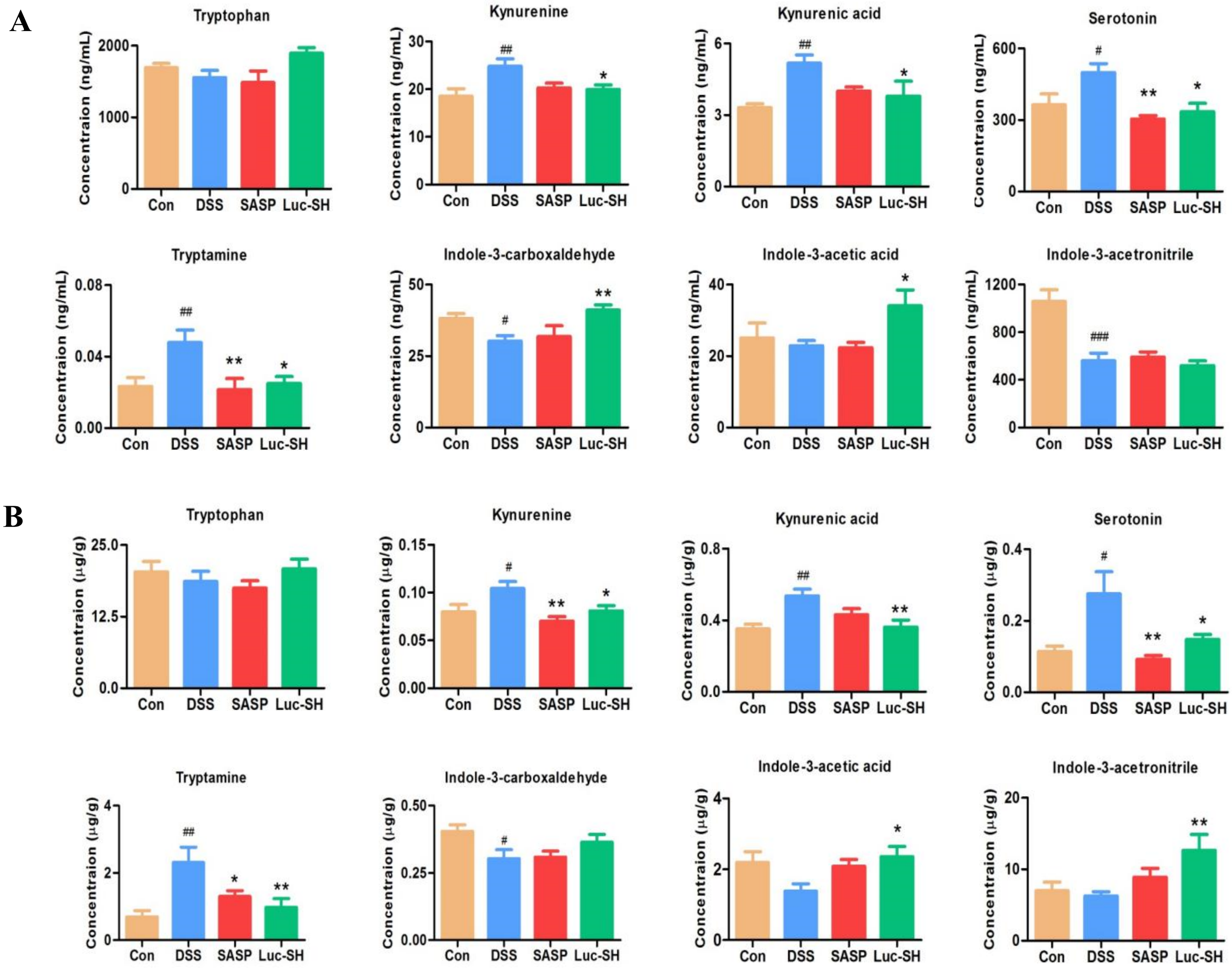

Fig S7 Fuc-S modulated host-microbe tryptophan metabolism of DSS-induced chronic colitis in mice. (A) The changes of tryptophan, kynurnine, kynurenic acid, serotonin, typtamine, indole-3-acetic acid, indole-3-acetronitrile and indole-3-carboxaldehyde in the serum. (B) The changes of tryptophan, kynurnine, kynurenic acid, serotonin, typtamine, indole-3-acetic acid, indole-3-acetronitrile and indole-3-carboxaldehyde in the feces. Results are expressed as mean ± SEM. (n=7-9) and analyzed using ANOVA followed by Duncan's multiple range tests. $^{\#}p<0.05$, $^{\#\#}p<0.01$ and $^{\#\#\#}p<0.001$, compared to Con group; $^{*}p<0.05$, $^{**}p<0.01$ and $^{***}p<0.001$, compared to DSS group. Con: control, SASP: salazosulfadimidine, Fuc-SH: Fuc-S with high dosage (200 mg/Kg).

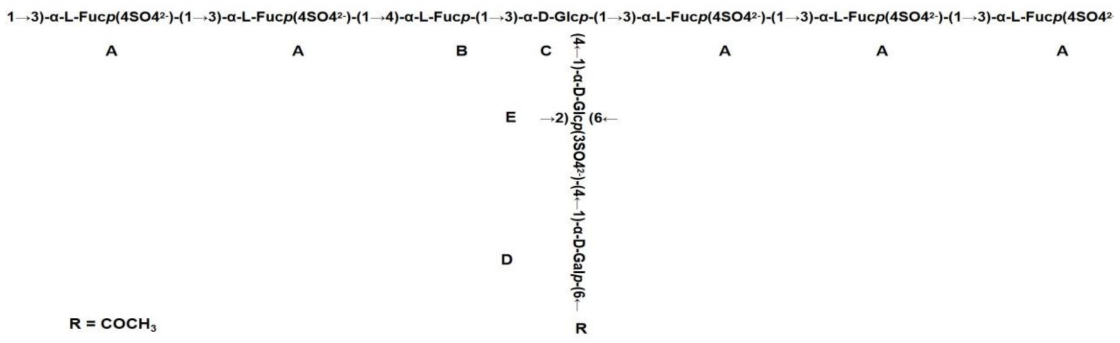

Fig S8 Predicted structure of Fuc-S.

2. Supplementary Table

Supplementary Table S1. The MRM transition for quantification tryptophan metabolites.

Compound Name	Prec Ion	Prod Ion	Frag (V)	CE (V)	Polarity
L-Kynurenine	209.1	192.1	50	5	Positive
L-Kynurenine	209.1	94	50	9	Positive
L-Tryptophan	205.1	188	90	5	Positive
L-Tryptophan	205.1	118	90	29	Positive
Kynurenic acid	190	144	110	17	Positive
Kynurenic acid	190	89	110	45	Positive
Serotonin	177.1	160	90	9	Positive
Serotonin	177.1	115	90	29	Positive
3-Indole-acetic acid	176.1	130	110	13	Positive
3-Indole-acetic acid	176.1	77	110	41	Positive
Tryptamine	161.1	144	50	9	Positive
Tryptamine	161.1	115.1	50	37	Positive
Indole-3-carboxaldehyde	146.1	118.1	110	13	Positive
Indole-3-carboxaldehyde	146.1	91.1	110	33	Positive
